# ADHD prescription patterns and medication adherence in children and adolescents during the COVID-19 pandemic in an urban academic setting

**DOI:** 10.1186/s12888-024-05623-4

**Published:** 2024-03-07

**Authors:** Peter J. Cunniff, Amil Ahsan, Catherine McCrary, Tracy Dien, Tristan H. Kuhn, Turaj Vazifedan, John W. Harrington

**Affiliations:** 1 Eastern Virginia Medical School, Norfolk, VA USA; 2 Department of Pediatrics, General Academic Pediatrics, Children’s Hospital of The King’s Daughters, Norfolk, VA USA

**Keywords:** ADHD, COVID-19, Telehealth, Telemedicine, Adherence, Pediatric, Race

## Abstract

**Background:**

COVID-19 impacted all students, especially those with attention deficit hyperactivity disorder (ADHD), putting them at risk for disruption to their medication regimen and school performance. Our study aimed to identify if ADHD medication regimens were disrupted through analyzing prescription refills and if telehealth management demonstrated a higher rate of adherence.

**Methods:**

A total of 396 patients from the General Academic Pediatrics (GAP) clinic at Children’s Hospital of The King’s Daughters (CHKD) were included in the study. Patients were between the ages of 8–18 with a history of ADHD for three or more years that was medically managed with four or more prescription refills between January 2019 and May 2022. A retrospective chart review collected age, sex, race, refill schedule, appointment schedule, and number of telehealth appointments. Data analysis compared the variables and defined “pre-pandemic months” as January 2019 through March 2020 and “pandemic months” as April 2020 through June 2022.

**Results:**

The total percentage of patients who had their ADHD medications during pre-pandemic months ranged from 40 to 66% versus 31–44% during pandemic months. Additionally, the total percentage of patients who had quarterly ADHD management appointments during pre-pandemic months ranged between 59 and 70% versus 33–50% during pandemic months. The number of months with ADHD prescription refills over the last three years was significantly higher among those who had both virtual and in-person visits than those who had just in-person visits, *p* < 0.001. Regarding race, Black patients had a lower number of medication refills compared to White patients when controlled for appointment type. They also had a lower number of total appointments, but there was not a significant difference in the number of virtual appointments.

**Conclusions:**

Since the start of the pandemic, ADHD patients have both refilled their prescriptions and returned to clinic less frequently. This data suggests a need to re-evaluate the ADHD symptoms of GAP patients periodically and return them to a more consistent medication regimen. Telehealth appointments are a potential solution to increase adherence. However, racial inequities found in this study need to be addressed.

**Supplementary Information:**

The online version contains supplementary material available at 10.1186/s12888-024-05623-4.

## Background

COVID-19 altered the traditional classroom education model from an in-person environment to almost exclusively virtual and hybrid platforms, which placed students with attention deficit hyperactivity disorder (ADHD) at risk for disruption to their education. ADHD is defined by poor attention, distractibility, hyperactivity, impulsiveness, or behavioral problems [1]. During months of remote learning, schools had mixed results in student performance, with both improvement and worsening in mathematics and reading [2, 3]. Students with neurodevelopmental disorders, however, struggled the most, and students with ADHD had difficulty focusing during online classes [2]. In addition, adolescents with ADHD were significantly more likely to have a parent report remote learning as "very challenging" than adolescents without ADHD, regardless of whether they had an individualized educational plan (IEP) or 504 plan, which provide school accommodations with or without specialized instruction and guidelines for students with documented disabilities [4, 5]. Even without the added stressors of the pandemic, children and adolescents with ADHD were particularly susceptible to changes to their education platform because their IEPs, medication routines, and personal schedules were designed specifically for traditional in-person education, not remote or hybrid learning.

In patients over five years of age, pharmacologic management with behavioral accommodations is the first line treatment for ADHD in the United States [6]. Patients with untreated ADHD may suffer from impaired school performance and are at higher risk of entirely withdrawing from school [7]. When treated pharmacologically, children have significantly improved school performance, long-term work ethic, and social outcomes [1]. Monitoring ADHD, however, proved challenging during the pandemic due to variable learning schedules that regularly changed. Students with ADHD who were doing remote learning during COVID-19 lockdowns had difficulty completing school assignments and had increases in inattention, impulsivity, and aggression [8, 9]. On the other hand, they also had improvements in anxiety and self-esteem, which parents attributed to decreased negative feedback at school and more flexible schedules at home [8]. Because patients’ experiences with ADHD constantly changed during the pandemic, they may have had a greater need to regularly follow-up with physicians for ongoing management.

Unfortunately, the pandemic also disrupted medication compliance and ADHD management. U.S. President Donald Trump declared COVID-19 a national emergency on March 13, 2020 [10]. According to insurance claims, drug-dispensing totals for pediatric patients from April to December 2020 were 27.1% lower compared to April to December 2019, and ADHD medication dispensing dropped by 11.8% [11]. Another study found that there was a 2.84% decrease in dexmethylphenidate refills between March and August 2020 [12]. Regarding COVID-19’s impact on outpatient care, England saw a 23.5% drop in the number of outpatient appointments for patients under 25 years of age between March 2020 and February 2021, which occurred despite a large rise in phone appointments [13]. In a different survey, half of pediatricians stopped evaluating new patients with ADHD, and only 5% still offered in-person services for ADHD management [14]. Overall, the decrease in stimulant use and physician management, on top of COVID-19 lockdowns and remote learning, put children with ADHD at greater risk for symptom exacerbation and its educational consequences.

All ADHD management was recommended to continue via telephone or a virtual platform when the pandemic began in accordance with American Psychiatric Association telepsychiatry guidelines, and this change brought about an unprecedented demand in telehealth [15]. A pediatric center in Washington, D.C. had 1,654 telehealth encounters between January 2016 and March 2020, but between March and June 2020, that number increased to 45,236 [16]. England experienced 2.6 million more phone appointments for patients under 25 years of age between March 2020 and February 2021 than the previous three years [14]. Despite this shift in appointment type, past studies evaluating ADHD telehealth management demonstrated consistent quality of care when compared to in-person management [17, 18]. A review from Current Problems in Pediatric and Adolescent Health Care reported 95–100% adherence to the American Academy of Pediatrics assessment guidelines from 2000 when diagnosing ADHD via a telehealth platform, while acknowledging that further evaluation is needed when using the updated AAP guidelines [17]. Regarding pharmacological and behavioral therapies, telehealth had comparable results to traditional, in-person care when evaluating for improvements in child behavior, symptoms, and functional outcomes [17]. The Children’s ADHD Telemental Health Treatment Study (CATTS) even saw improvements in symptoms compared to standard, in-person care when patients received pharmacotherapy over videoconferencing in conjunction with in-person parental behavior training [18]. Lastly, patients have found telephone and virtual ADHD services to be effective and satisfactory, although many of them still prefer in-person management [19]. This is consistent with satisfaction surveys evaluating other telemedicine services, including obesity, asthma, other mental health conditions, and subspecialty appointments [20]. Thanks to telehealth’s demonstrated effectiveness, satisfaction rates, and the sudden demand for distanced care, it became an alternative means for physicians to manage ADHD.

Even though there are existing studies that evaluate the impact of COVID-19 on ADHD management and the effectiveness of telehealth in treating ADHD, we found little to no data comparing the two during the COVID-19 era upon literature review. On one hand, patients experienced worsening ADHD symptoms during lockdown after the pandemic began, and fewer total in-person appointments were available [8, 11–14]. Conversely, telehealth appointments rapidly grew in number and had historically been underutilized but successful at managing ADHD [15–20]. Furthermore, while several studies examined ADHD symptoms and medication use during lockdown, few continued to follow these variables during the subsequent school years, during which students experienced changes between remote, in-person, and hybrid learning depending on local guidelines and infection rates. In our study, we compared ADHD management throughout the pandemic with pre-pandemic care by quantifying prescription refills and appointment schedules. We also used medication adherence to evaluate the effectiveness of incorporating telehealth into ADHD management during the pandemic. Our goals were to (1) identify the number of patients that discontinued medication management before and during the COVID-19 pandemic between January 2019 and June 2022 and (2) identify trends, if any exist, in ADHD medication demand using prescription refill data during that time related to race, appointment type, and timing relative to the pandemic. We hypothesized that there would be a decrease in the number of refills after the pandemic reached the United States in March 2020, a slow increase starting in September 2020, and a return to baseline refill rates by September 2021. We also expected a higher number of refills among patients with telehealth encounters over patients who were only managed in-person.

## Materials and methods

A retrospective chart review collected age, sex, race, monthly refill schedule, and quarterly appointment schedule of patients followed by the General Academic Pediatrics (GAP) department of the Children’s Hospital of The King’s Daughters (CHKD). The CHKD GAP clinic is known for being a safety net clinic in Norfolk, VA, with the vast majority of its patients on Medicaid, a US Government sponsored health insurance program for lower income patients, or Medicaid managed care. The practice sees about 23,000 patients annually and is the primary teaching outpatient clinic for pediatric residents and medical students at Eastern Virginia Medical School. Patients were selected if they were between the ages of 8–18, had an ICD-10 diagnosis code of ADHD confirmed before January 2019, and had an ADHD medication refilled a minimum of four separate months between January 2019 and May 2022. A patient’s refill schedule was determined by whether they had an ADHD medication refilled at least one time per month between January 2019 and May 2022, and refills were dispensed in 30-day supplies, with the ability to call for renewals for two additional months, per GAP clinic policy. Similarly, the quarterly appointment schedule was determined by whether a patient was seen and managed for ADHD at least one time during defined three-month periods (January-March, April-June, July-September, and October-December) between January 2019 and May 2022. Appointments were considered ADHD management appointments if the physician’s note described the patient’s current ADHD symptom management and medication use. Telehealth appointments had been available to patients at their or the physician’s request if they had been seen in-person at the GAP clinic within the last year. Research assistants were able to search our database for active patients with the ICD-10 codes specific for ADHD and then reviewed the GAP clinic electronic medical record, Cerner Millennium ®. They recorded age, sex, race, monthly refill schedule, and quarterly appointment schedule into a secure, password protected REDCap database, and deidentified the patient data. Data analysis compared the variables and defined “pre-pandemic months” as January 2019 through March 2020 and “pandemic months” as April 2020 through June 2022. Continuous variables were presented as mean, standard deviation, median, Min, and Max. Categorical variables were presented as frequency and percentage. Pearson correlation was used to assess the association between continuous variables. T-test and Man-Whitney tests were used to analyze differences in the number of appointments between White and Black populations. Generalized Liner Model was used to assess the impact of race and type of appointments on the percentage of total refills. All statistical tests were performed using SPSS.26 (Chicago, IL). All statistical tests were two-sided, and *p* < 0.05 was considered statistically significant. Variables were compared using either two-sample t-tests or linear regression. Values of 1 > *r* ≥ 0.8 were considered strong positive linear correlation, 0.8 > *r* ≥ 0.4 moderate positive linear correlation, 0.4 > *r* > 0 weak positive linear correlation, *r* = 0 no correlation, 0 > *r*≥-0.4 weak negative linear correlation, -0.4 > *r*≥-0.8 moderate negative correlation, and − 0.8 > *r* ≥ 1 strong negative correlation.

## Results

**Fig. 1:**
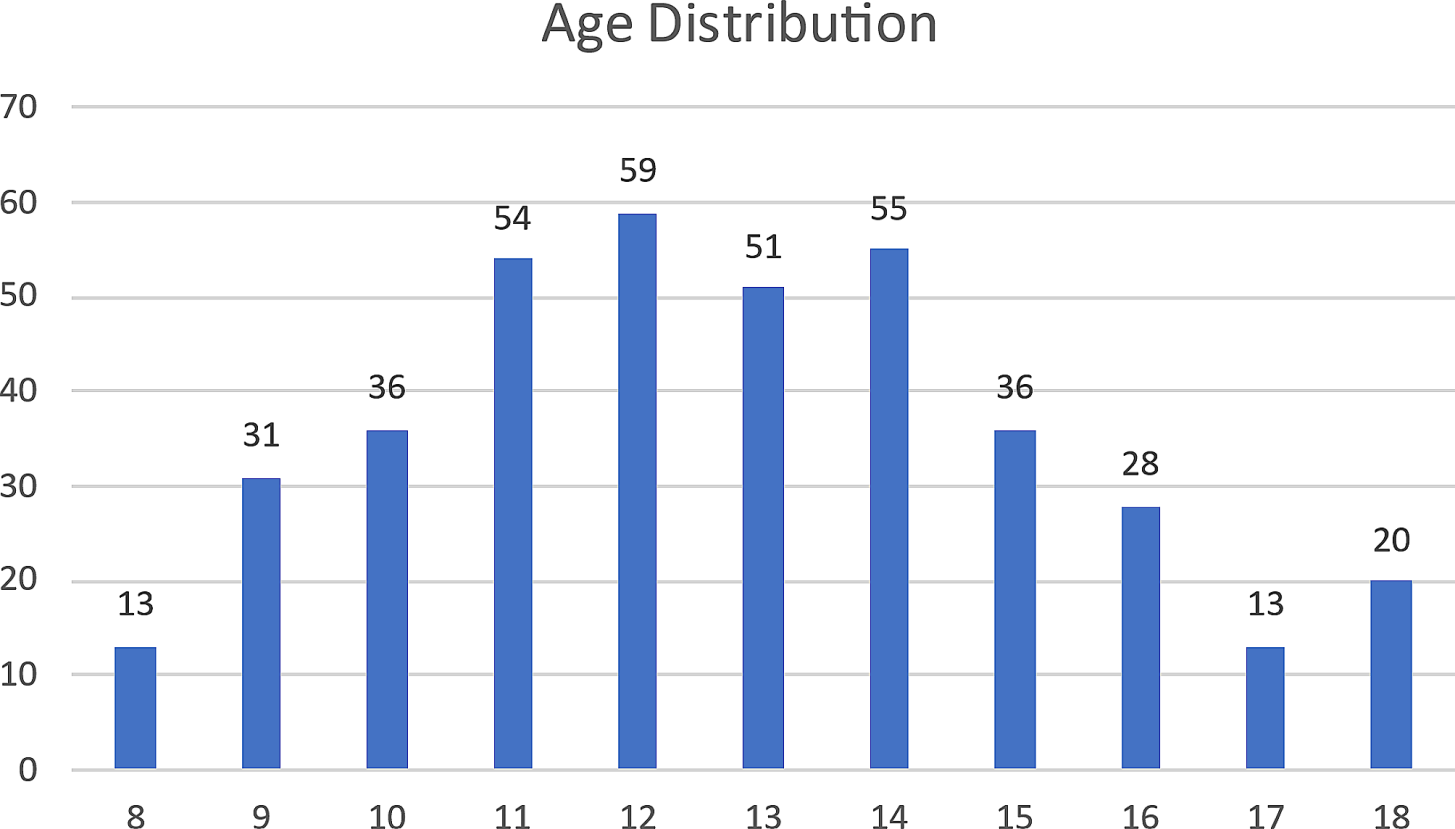
The age distribution of the patients

Data was obtained from 475 patients. Seventy-nine were removed because they either refilled their medication three or less times or did not have a complete prescription history available during the 41-month period. Of the 396 charts utilized, 302 patients were male and 94 were female. Additionally, 314 patients identified as Black, 78 as White, one as Asian, and 3 as not disclosed, which mirrored our overall patient demographic (Fig. 1; Table 1).

**Table 1:** The demographic information of the participants

		Count	Column N %
Sex	Male	302	76.3%
	Female	94	23.7%
Race	White	78	19.7%
	Black or African American	314	79.3%
	American Indian or Alaska Native	0	0.0%
	Asian	1	0.3%
	Native Hawaiian or Other Pacific Islander	0	0.0%
	Not disclosed	3	0.8%

**Fig. 2:**
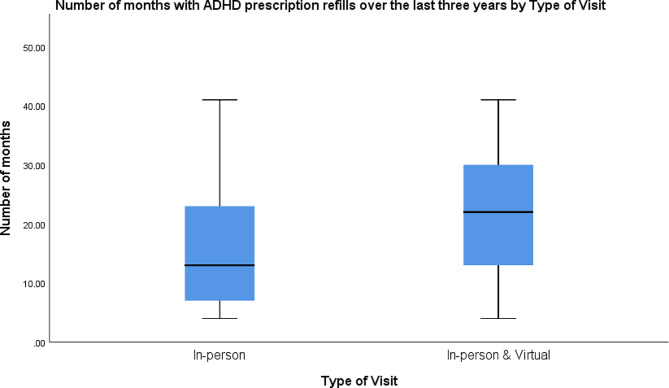
The distribution of the total number of months with ADHD prescription refills between January 2019 and May 2022 in relation to appointment type

**Fig. 3:**
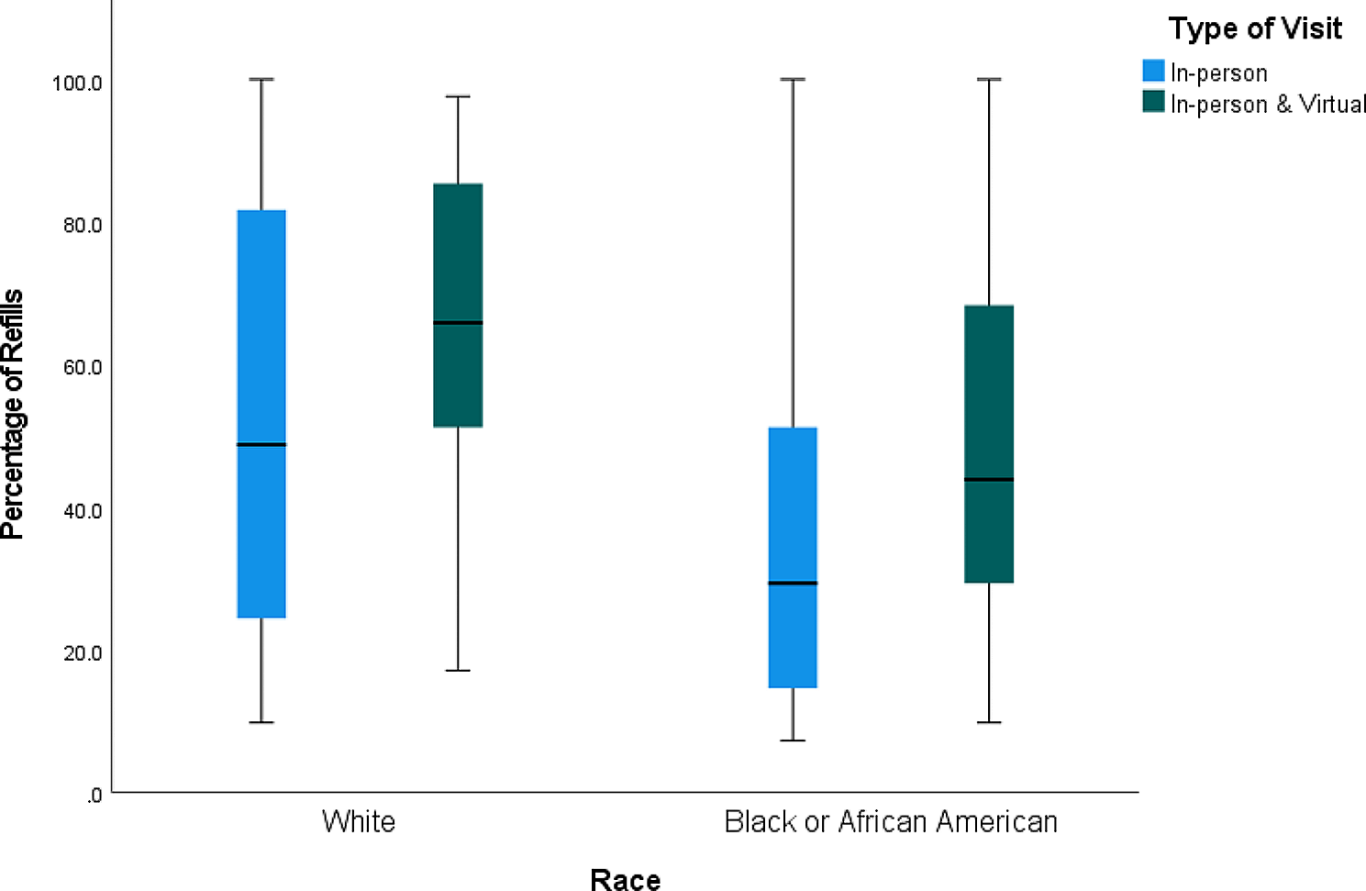
The distribution of the percentage of months with ADHD prescription refills between January 2019 and May 2022 in relation to race and appointment type

**Fig. 4:**
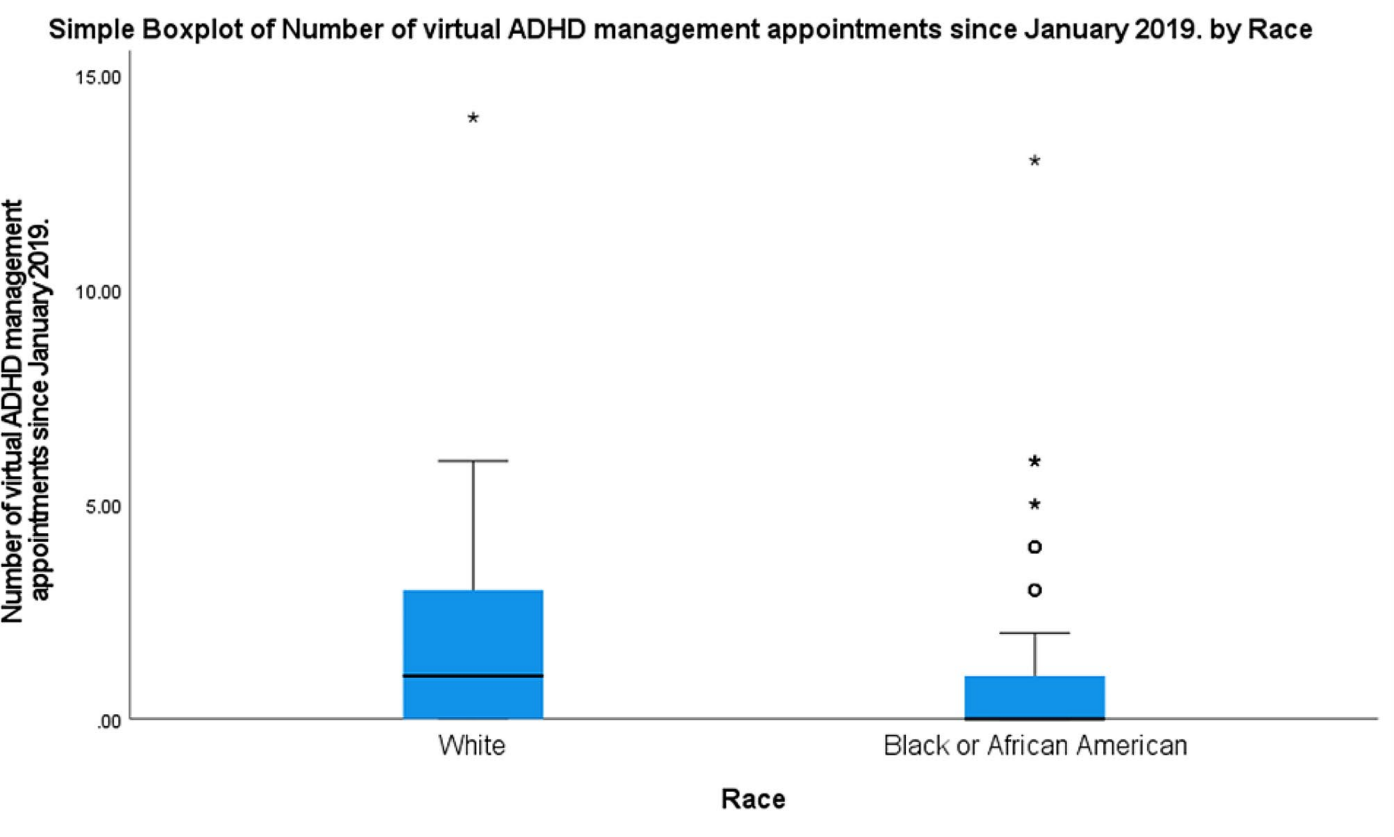
The distribution of the number of ADHD management appointments in relation to race

**Fig. 5:**
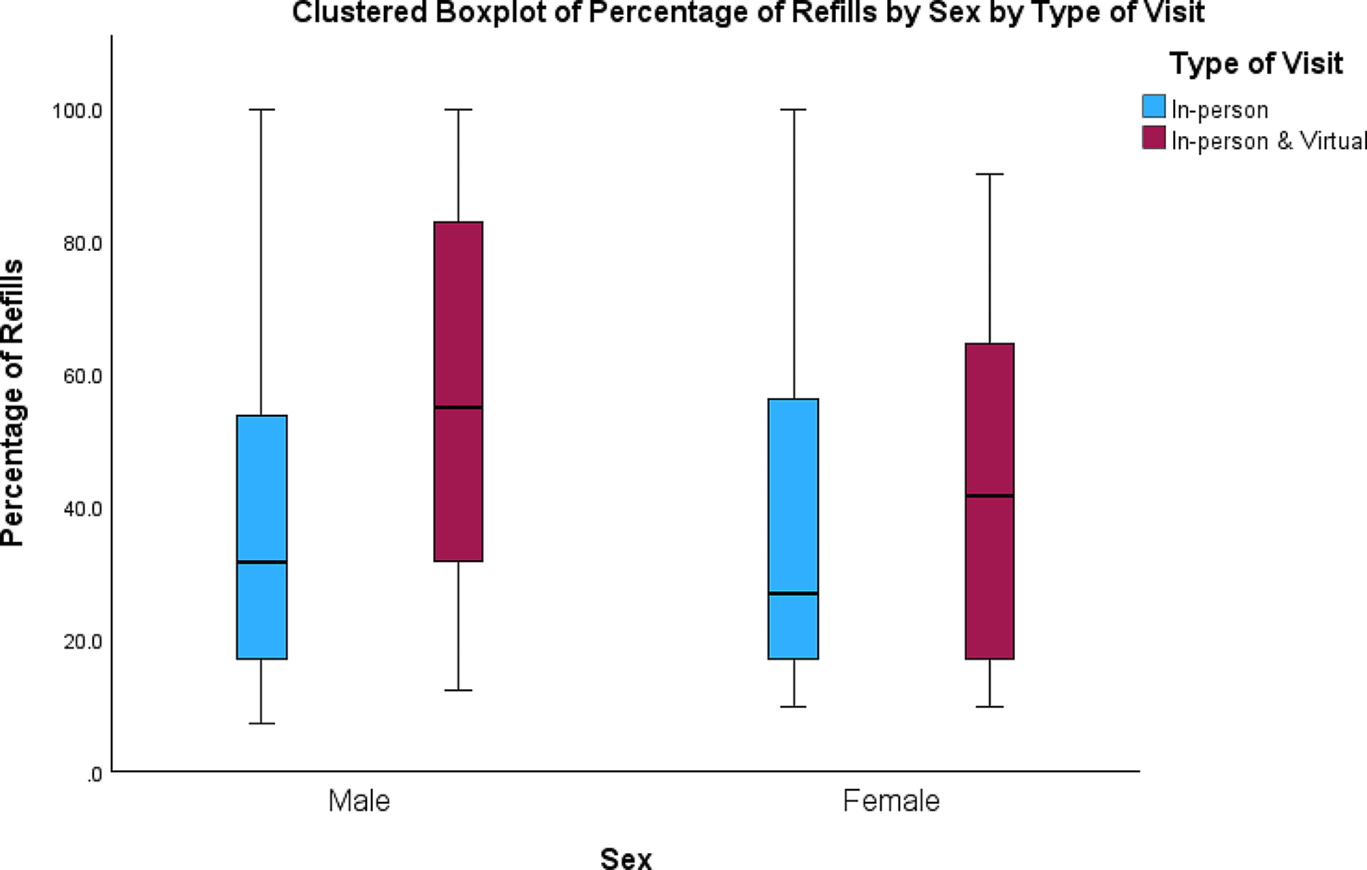
The distribution of the total number of refills in relation to sex and appointment type

The number of months that ADHD prescription was refilled was significantly correlated with total number of ADHD appointments (*r* = 0.40, *p* < 0.001), number of virtual (*r* = 0.27, *p* < 0.001), and number of in-person (*r* = 0.35, *p* < 0.001) ADHD appointments. The number of months (m) with prescription refills over the last three years was significantly higher among those who had both virtual and in-person visits (m = 22.03) than those who had just in-person visits (m = 15.97) (*p* < 0.001) (Fig. 2). This remained significant when accounting for racial background. Black patients with both in-person and virtual appointments had a higher number of refills than Black patients with only in-person appointments (m = 20.2 vs. 15.1) (*p* < 0.001), and the same was true for White patients (m = 26.6 vs. 21.2) (*p* = 0.022) (Fig. 3). Regarding racial differences, the number of virtual appointments was significantly lower among all Black patients (m = 0.7) than all White patients (m = 1.6) (*p* < 0.001), but there was not any significant difference in the total number of appointments (*p* = 0.08) and number of in-person appointments (*p* = 0.88) between White and Black patients (Fig. 4). The number of refills was also significantly higher among White patients who had only in-person appointments or both in-person and virtual appointments than Black patients with the same type of appointments (m = 21.2 vs. 15.1; m = 26.6 vs. 20.2) (*p* = 0.001; *p* < 0.001) (Fig. 3). Regarding age, there was a negative correlation between age and number of months that ADHD prescriptions were refilled (*r*=-0.12, *p* = 0.012). Also, age was negatively correlated with total number of ADHD appointments (*r*=-0.30, *p* < 0.001) and number of in-person appointments (*r*=-0.31, *p* < 0.001). There was not any significant association between age and virtual appointments (*r*=-0.05, *p* = 0.29). Regarding sex, the percentage of refills among males who had in-person & virtual appointments was significantly higher than males with only in-person appointments (m = 23.1 vs. 16) (*p* < 0.001) and females with both in-person & virtual appointments (m = 23.1 vs. 18.0) (*p* = 0.011) (Fig. 5).

**Fig. 6:**
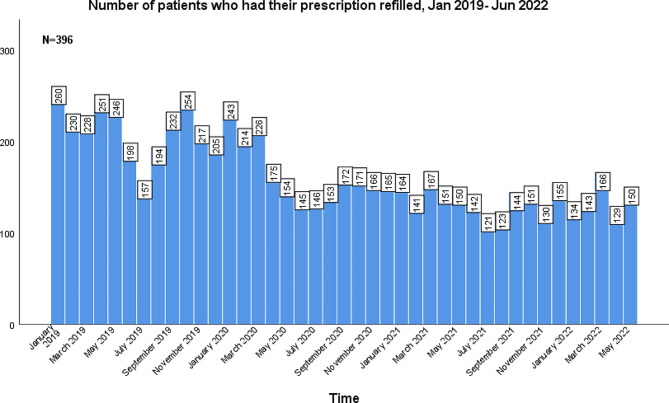
The number of patients that refilled their ADHD prescription in a given month between January 2019 and May 2022

**Fig. 7:**
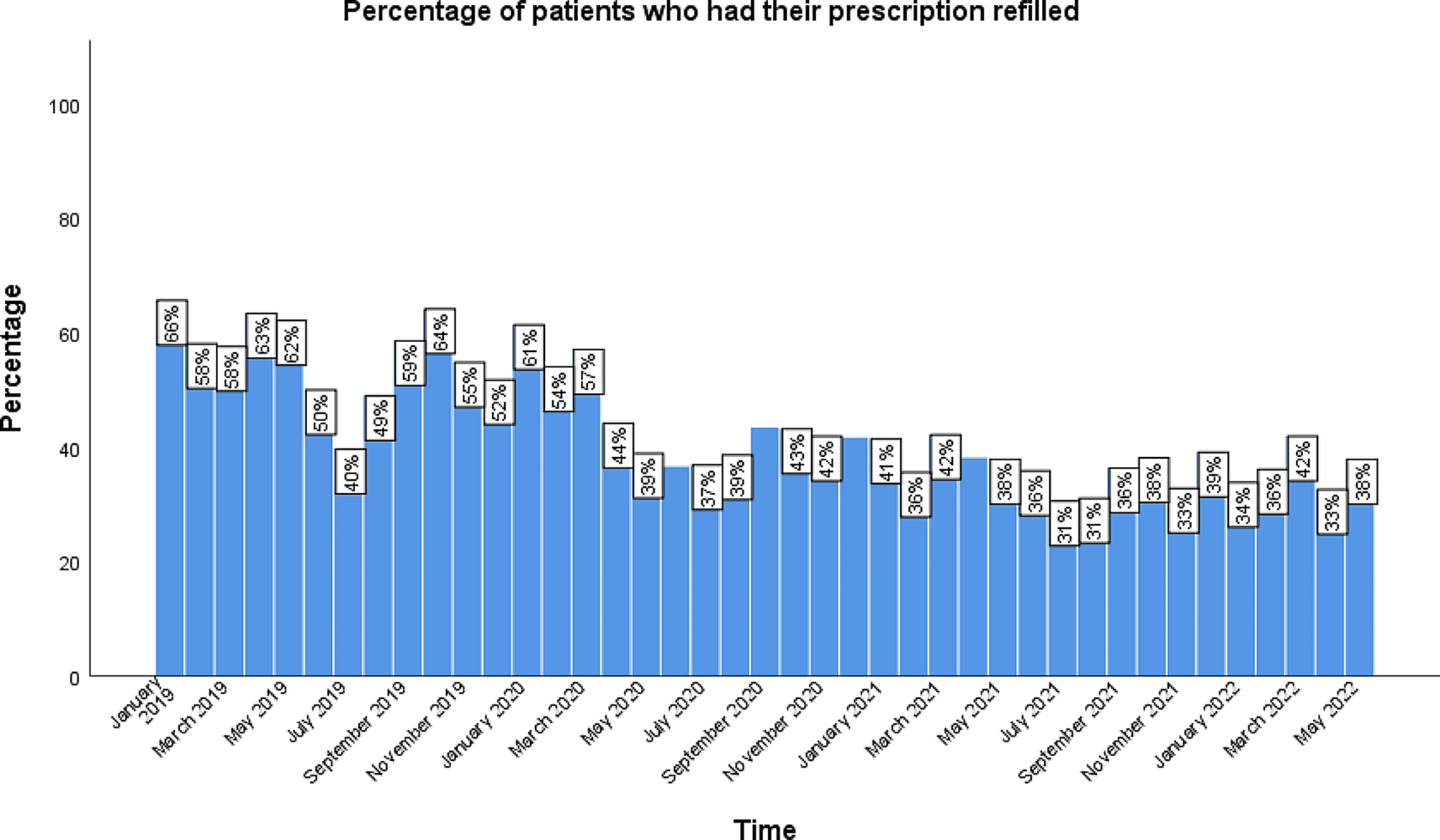
The percentage of patients that refilled their ADHD prescription each month between January 2019 and May 2022

The months with an ADHD prescription refill were divided into two groups, pre-pandemic and pandemic, defined in the methods. The total number of patients who had an ADHD prescription refilled in a given month during the pre-pandemic months ranged from 157-260 (40–66%) as opposed to 121–175 patients (31–44%) during the pandemic months (Figs. 6 and 7). There was a significant decrease in patients refilling their prescriptions each month during the pandemic compared to the pre-pandemic (*p* < 0.001).

**Fig. 8:**
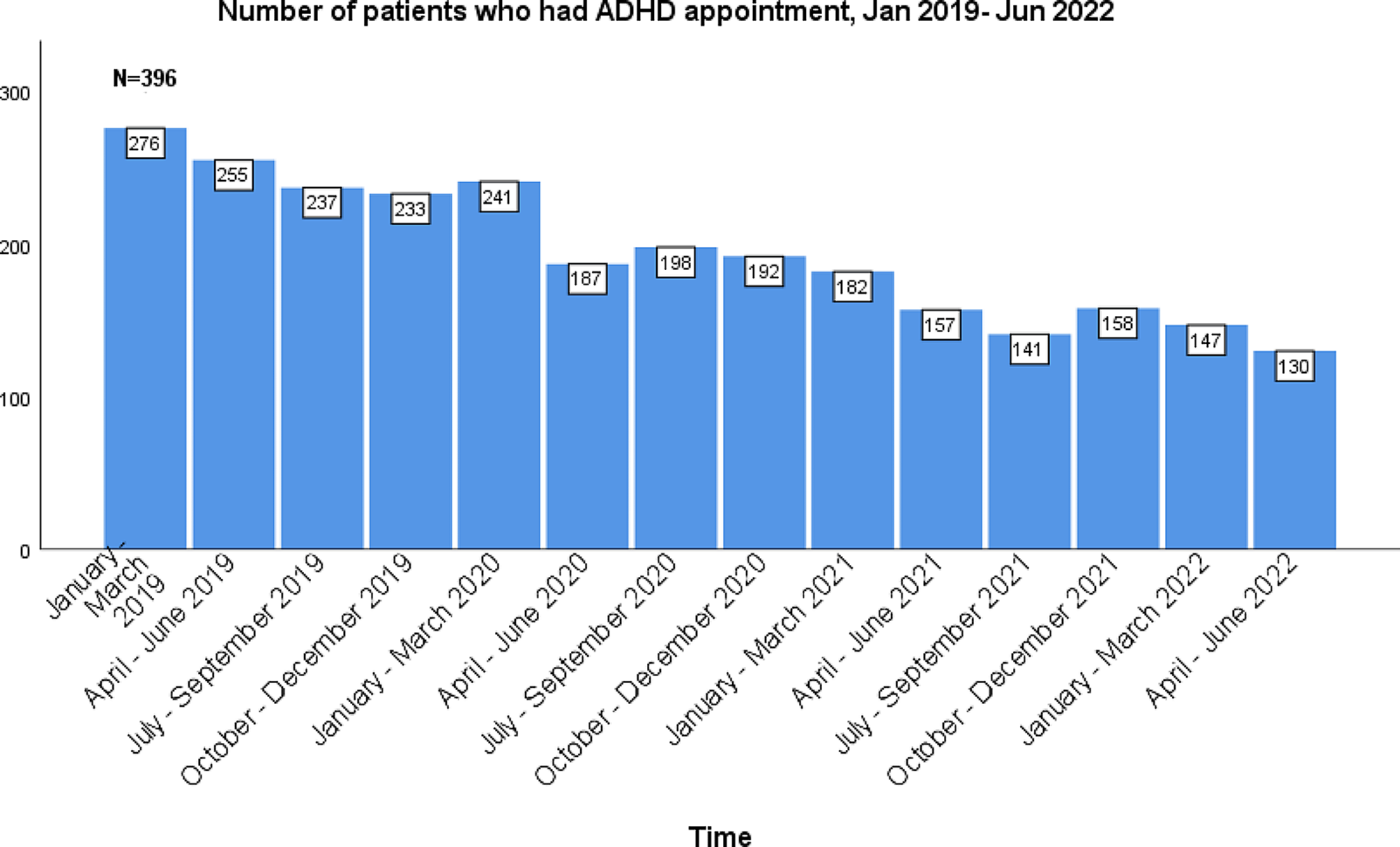
The number of patients who had an ADHD management appointment by quarter

**Fig. 9:**
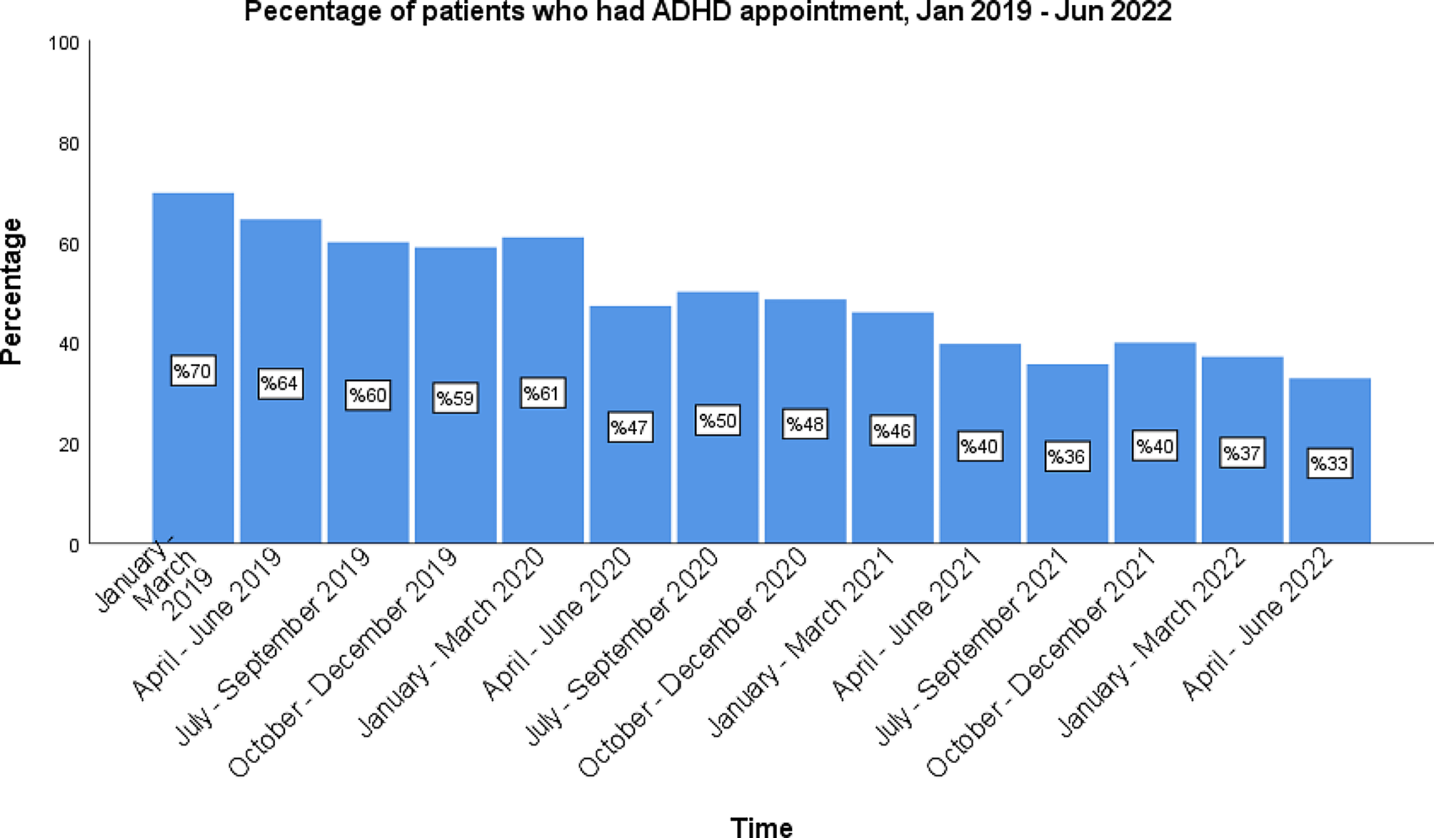
The percentage of patients who had an ADHD management appointment by quarter

The quarterly appointment schedule was similarly divided into pre-pandemic and pandemic groups. The total number of patients who had quarterly ADHD management appointments during the pre-pandemic months ranged between 241-276 (59–70%) as opposed to 130–198 patients (33–50%) during the pandemic months (Figs. 8 and 9). There was a significant decrease in the number of patients with quarterly appointments during the pandemic months (*p* < 0.001).

## Discussion

Our study evaluated the medication adherence of patients with ADHD from one year before the pandemic to over two years into the pandemic. First, we found that medication refills decreased significantly after March 2020, and each pandemic month had a lower refill rate than any pre-pandemic month (31–44% versus 40–66%) (*p* < 0.001), except for July 2019. The decrease in medication refills between June and August 2019 was expected because many patients often do not take medications during the summer when they are not attending school [21]. These findings are consistent with reports from parents of patients with ADHD that their children had worsening ADHD symptoms during lockdown and challenges with remote learning [4, 8]. Because ADHD medication use relies on a regular daily schedule, such as taking the medication after breakfast or at certain times during the school day, the new dynamic of a remote learning environment during lockdown and COVID-19 surges were not conducive to maintaining a predictable schedule. Regularly changing school schedules necessitated regularly changing medication schedules, which could easily lead to missed doses or discontinuing medications entirely. Additionally, if some were required to stay home and in closer proximity to their children during remote learning, they potentially could provide more individual attention. This increased attention and vigilance may have allowed some children to stay on task with schoolwork for a longer time with less reliance on medications [22]. 

We expected refill rates to increase back to baseline by the start of the 2021/2022 academic year as schools across the country transitioned back to full-time in-person learning, but they remained unchanged. These patients did not necessarily find other coping strategies for their ADHD. While there is little published research examining ADHD symptoms and school performance after the COVID-19 lockdowns, the National Assessment of Educational Progress reported significant test score declines among fourth and eighth grade students in math and reading between 2019 and 2022 [23]. In Virginia specifically, Standards of Learning test pass rates in reading, math, and science were lower in 2021/2022 than 2018/2019 [24]. Meanwhile, 41.4 million Adderall prescriptions were dispensed in 2021, a 10–20% increase from 2020, which is the opposite of what we found among our patients [25, 26]. Even before the pandemic started, only 40–66% of patients had prescription refills in a given month. Students with ADHD are already at risk for low adherence given the nature of the condition, and adherence rates varied greatly [27]. One Canadian study from 2012 reported discontinuation rates of 19% with long-acting stimulants and 39% with short acting, and another study evaluating GPA improvement with stimulant adherence among Philadelphia public school students only had a 20% adherence rate [27, 28]. The prognosis of ADHD typically extends from childhood into adolescence, and even adolescents who had responded well to treatment usually continue to have significant impairment that extends into adulthood [6, 29]. In our study, we saw a slight negative correlation between age and number of months that an ADHD prescription was refilled (*r*=-0.12, *p* = 0.012), which cannot solely account for the difference in pre-pandemic and pandemic refill rates. We therefore think the unchanged refill rate is less likely due to an improvement in patients ADHD symptoms but rather other factors preventing our patients from adhering to their regimen. In the context of the pandemic, COVID-19 seemed to exacerbate ADHD symptoms and medication non-compliance, and our patient population was not able to recover to pre-pandemic levels.

Predictors that favor ADHD medication adherence include two-parent families, higher socioeconomic status, and Caucasian background [30]. The GAP clinic is a safety net clinic that primarily serves Black patients on Medicaid. This differential impact arises from the cost of the medication, added financial stressors during the pandemic, and supply chain issues that could prevent a family from refilling an ADHD medication. In our study, 79.3% of the subjects were Black, so our population correlated with a limited rate of adherence that was sustained. In addition to the low monthly pre-pandemic refill rates among all our patients, Black patients had a significantly lower number of months with refills than White patients with the same type of appointment (*p* < 0.001). Distrust in the medical system is also associated with decreased adherence [30]. In the context of misinformation about COVID-19 and vaccine safety, another study at the GAP clinic surveyed the parents of pediatric patients and found that 67.5% of 179 respondents were unlikely to vaccinate their child, with 73.1% of Black parents, or 87 parents, reporting "unlikely to vaccinate" [31]. This hesitancy could also foster mistrust in other aspects of the patients’ healthcare, including ADHD management. Other negative associators of ADHD medication adherence are lack of early follow-up after starting treatment and limited transportation services, while a good patient-physician relationship was associated with increased adherence [32]. In the context of our study, the patients who had the greatest number of refills returned to clinic the most (*r* = 0.40, *p* < 0.001). However, we also saw only 59–70% of ADHD patients return for follow-up every three months before the pandemic and 33–50% during the pandemic. The Healthcare Effectiveness Data and Information Set recommends two follow-up appointments after the first month of initiating an ADHD medication, one at three months and another at six months, so our patient follow-up rates were below the recommended guidelines [33]. As a whole, our population of patients already had several socioeconomic factors that limited their ability to engage with their healthcare and refill their medications prior to the pandemic.

Incorporating telehealth into follow-up plans may be beneficial to increasing medication adherence. Having at least one telehealth management appointment correlated with a greater number of total months with refills, with a median of 27 versus 21 months among White patients (*p* = 0.022) and 18 versus 12 months among Black patients (*p* < 0.001) over a period of 41 months. These results are comparable to the CATTS findings that demonstrated telehealth as a successful means to monitor ADHD symptoms and other studies showing improved follow-up rates when using telehealth [18, 34, 35]. Furthermore, integrating technology into patient care has already been successful in improving medication adherence. One study evaluated the efficacy of using a mobile app to track ADHD symptoms, provide medication reminders, and facilitate patient-physician communication and found that patients using the app took their medication more regularly than the control group [36]. Similar results have been found among other groups including hypertension and diabetes patients [37, 38]. In our patient population, White patients had on average 1.6 virtual appointments while Black patients only had 0.7 (*p* < 0.001). There was a trending towards significance in the total number of appointments (*p* = 0.08) but no significant difference in the number of in-person appointments (*p* = 0.88) between White and Black patients. The difference in the number of telehealth appointments could be due to a lack of reliable smartphone, computer, or Wi-Fi access among our Black patients. Another possibility is that the patients able to do a combination of visit types may have highly organized, resourceful parents or fewer barriers to healthcare, which would also lead to a higher rate of prescription refills. Regardless, telehealth has clear benefits in health management including improved access to appointments and reduced cost, which is especially beneficial for ADHD patients who may have difficulty with in-person appointments [39]. For our patient population at the GAP clinic, telehealth also could provide an opportunity for more appointments and patient education. This could increase patient engagement, which in turn leads to increased adherence as demonstrated by patients utilizing medical apps [36–38]. Improving access to telehealth and encouraging telehealth follow-ups, especially with our Black patients, thus could be conducive to increasing medication compliance among patients with ADHD.

We were limited in means to track medication adherence over the period of 41 months we analyzed. While refilling a medication shows intention to use it, patients might not have been taking their medications consistently or could have adjusted their doses without consulting their physicians. These possibilities would change the number of refills they needed due to a potential surplus of medication, so they may not have needed to refill their medication as frequently. Recording the number of months with prescription refills would catch the patients with missed or altered doses but not any patients who may refuse to take their medications even when their parents continue to refill it. Alternatively, surveying patients about their medication habits over three years could lead to inaccurate data due to recall bias. Therefore, analyzing prescription refills was the most reliable and efficient means to measure adherence. We elected not to evaluate the patients’ ADHD symptoms through the use of Vanderbilt© screening in relation to their patterns of refills, since this process was also disrupted significantly and would have been difficult to interpret. In addition, since CHKD GAP is a teaching clinic, numerous pediatricians manage our set of patients, and due to different styles of notetaking, we could not standardize symptoms into categories such as mild, moderate, or severe. Regarding differences between patients that did or did not utilize telehealth services, we did not have means available to evaluate for differences between the two populations, such as socioeconomic disparities, because that information was not available to us in the electronic medical record throughout this time.

This study quantified the impact and lingering effects of the COVID-19 pandemic on ADHD medication adherence in patients at the GAP clinic, and on a larger scale, identified a strong need to reevaluate symptoms and management among underrepresented youth with ADHD, especially Black patients. It also identified a positive correlation between incorporating telehealth into management and medication adherence. The CATTS study already saw an improvement in ADHD symptoms in rural patients with telehealth appointments compared to traditional in person care [18]. Future randomized clinical trials must be performed to determine whether the same is true for Black patients and patients from an urban setting, with close attention to medication use. Prospective studies could also investigate differences between patients with telehealth management versus in-person only management to highlight barriers to care patients might face. In any event, the pandemic worsened symptoms and adherence rates in children and adolescents already at risk for noncompliance, and there is an urgent need for pediatricians to reengage ADHD patients with their condition.

## Conclusion

The COVID-19 pandemic caused an unprecedented disruption to the medical management of children and adolescents with ADHD. Our study found that a significant decrease in prescription refills and follow-up appointments occurred when the pandemic started, which disproportionately affected Black patients, and never returned to pre-pandemic levels. Reasons for decreased adherence are multifactorial, but telehealth appointments are a potential, accessible solution to mitigating these factors. Even though the COVID-19 emergency is over in the US, there is a need for pediatricians to reengage patients with their ADHD management to recover from the pandemic’s lasting impact. Further prospective studies investigating differences between in-person follow-up and follow-up augmented with telehealth, including medication adherence, symptom management, and racial differences, are warranted.

### Electronic supplementary material

Below is the link to the electronic supplementary material.

Supplementary Material 1

## Data Availability

All data generated or analyzed during this study are included in this published article [and its supplementary information files].

## Abbreviations

ADHD: Attention deficit hyperactivity disorder
GAP: General Academic Pediatrics
CHKD: Children’s Hospital of The King’s Daughters
IEP: Individualized educational plan
CATTS: Children’s ADHD Telemental Health Treatment Study
